# Assessment of Landsat-8 and Sentinel-2 Water Indices: A Case Study in the Southwest of the Buenos Aires Province (Argentina)

**DOI:** 10.3390/jimaging9090186

**Published:** 2023-09-18

**Authors:** Guillermina Soledad Santecchia, Gisela Noelia Revollo Sarmiento, Sibila Andrea Genchi, Alejandro José Vitale, Claudio Augusto Delrieux

**Affiliations:** 1 Instituto de Ciencias e Ingeniería de la Computación, Universidad Nacional del Sur (UNS)-CONICET, San Andrés 800, Campus Altos del Palihue, Bahía Blanca 8000, Argentina; cad@uns.edu.ar; 2 Departamento de Ingeniería, Universidad Nacional del Sur UNS, Av. Além 1253, Bahía Blanca 8000, Argentina; 3 Instituto de Ecorregiones Andinas, Universidad Nacional de Jujuy (UNJU)-CONICET, Canónigo Gorriti 237, San Salvador de Jujuy 4600, Argentina; giselarevollo@gmail.com; 4 Facultad de Ingeniería, Universidad Nacional de Jujuy UNJU, Ítalo Palanca 10, San Salvador de Jujuy 4600, Argentina; 5 Instituto Argentino de Oceanografía (UNS)-CONICET, Florida 8000, Bahía Blanca 8000, Argentina; genchi.sibila@gmail.com (S.A.G.); vitale.alejandro@gmail.com (A.J.V.); 6 Departamento de Geografía y Turismo, Universidad Nacional del Sur UNS, 12 de Octubre y San Juan, Bahía Blanca 8000, Argentina; 7 Departamento de Ingeniería Eléctrica y de Computadoras, UNS, San Andrés 800, Campus Altos del Palihue, Bahía Blanca 8000, Argentina

**Keywords:** multi-spectral imaging, airborne imagery, normalized difference water index, geographical measurements

## Abstract

The accuracy assessment of three different Normalized Difference Water indices (NDWIs) was performed in La Salada, a typical lake in the Pampean region. Data were gathered during April 2019, a period in which floods occurred in a large area in the Southwest of the Buenos Aires Province (Argentina). The accuracy of the estimations using spaceborne medium-resolution multi-spectral imaging and the reliability of three NDWIs to highlight shallow water features in satellite images were evaluated using a high-resolution airbone imagery as ground truth. We show that these indices computed using Landsat-8 and Sentinel-2 imagery are only loosely correlated to the actual flooded area in shallow waters. Indeed, NDWI values vary significantly depending on the satellite mission used and the type of index computed.

## 1. Introduction

Remote sensing has a large number of applications, among which the most common is classification of land covers and analysis of the changes that occur therein through time. One of the most vital Earth resources is surface water, which is undergoing changes in time and space as a consequence of land use types and global warming [1]. Also, analyses of surface water provision are important as a means to determine the predisposition to flooding in a given region [2]. In this context, satellite imagery is a valuable tool for monitoring land use in general and occasional floods in particular. Remote sensing techniques are the most cost- and time-effective methods available to quantify and map flooded areas, taking into consideration the spatial and spectral resolution of the sensors and the periodicity of the takes. Optical sensors aboard the satellite constellations are designed to register specific spectral bands that allow the differentiation of flooded from non-flooded areas.

The variation in size of different water bodies, which undergo changes due to various factors, has been analyzed over multiple periods, using remote sensing and Geographical Information System (GIS) techniques in conjunction with field validation. Generally, the normalized difference water Index (NDWI) is used to rate changes in the water area [3]. In this regard, several studies have been conducted using remote sensing data to detect spatial and temporal changes in flooded areas, study their changes, and assess actual or potential flood damage in urban regions. The majority of the flood maps were developed from surface reflectance using MODIS (Moderate-Resolution Imaging Spectroradiometer) data given the short revisit times and the wide area coverage of this mission [4,5]. Studies carried out by Pekel et al. [6] developed an innovative method for detecting water bodies by means of colorimetric analyses. The methodology is generic and can thus be applied to sensors with similar bands with adequate reliability, but it has been devised to be applied only on continental scales. Although MODIS sensors provide near-global coverage twice daily, the pixel resolution ranges between 250 m and 1 km, so it does not have an optimal spatial resolution for use in small areas [7]. Arguably, the most extensively utilized data source for remote sensing are Landsat images, which are available (in different constellations and thus different sensors) from 1972 to the present, offering the longest continuous global record of the Earth’s surface. This record has gathered spectral information from the Earth’s surface, granting scientists the ability to assess land cover changes that can be traced and evaluated over a span of 50 years. This extended time-frame offers robust statistical significance in numerous studies. Despite this valuable information, Landsat-8 (L8) imagery has a moderate spatial resolution and a long revisit time [8]. This limits their applicability in various situations [1]. For instance, in the study of inland water bodies, the actual shape and size of relatively small water bodies (with areas ranging between 1 m^2^ and about 5 ha [9]) is difficult to assess. This is due to the mixed pixel phenomenon that arises when a pixel footprint over the surface covers a varying proportion of water [10]. To address these limitations and improve land cover monitoring and change-detection mapping, the Sentinel-2 (S2) mission may be employed [11]. This mission provides a finer spatial resolution and shorter revisit times, which makes it more adequate for land cover monitoring and associated applications, such as deforestation studies, monitoring and modelling climate-induced changes, etc. [12,13,14], allowing smaller water bodies to be thoroughly studied [15,16].

Lakes and lagoons have been a relevant research subject, which in particular require water quality monitoring at different scales [17]. Specifically in Argentina, during late 2018 and early 2019, infrequent heavy precipitation led to an unusual increase in the size of lakes and small water bodies, and also a portion of otherwise productive areas becoming flooded. Even though large areas were flooded or damp in specific departments of the Buenos Aires province, the NDWI indices provided by satellite missions (especially MODIS) consistently overestimated the actual proportion of flooded areas, sometimes by significant percentages. An adequate assessment of the actual impact of the condition was frequently required by crop producers, governmental agencies and other actors, but in this particular context, the adequacy of remote-sensing-based information appeared to be of little use. For this reason, in this study, we attempt to determine the bias and variance in the determination of actual NDWI products using several indices (Xu, Gao, McFeeters) computed from Landsat-8 and Sentinel-2 imagery. We used airborne images as ground truth, in which the very high resolution allows determination of the actual proportion of dry and wet surface in a satellite pixel footprint, and thus the study of the actual influence of the mixed pixel effect of different water indices. We chose a typical water body in the Southwest of the Buenos Aires Province as a specific case of study, which makes our results a feasible reference for other similar water bodies along the entire geographical region.

## 2. Materials and Methods

### 2.1. Study Area and Sample Preparation

**Figure 1:**
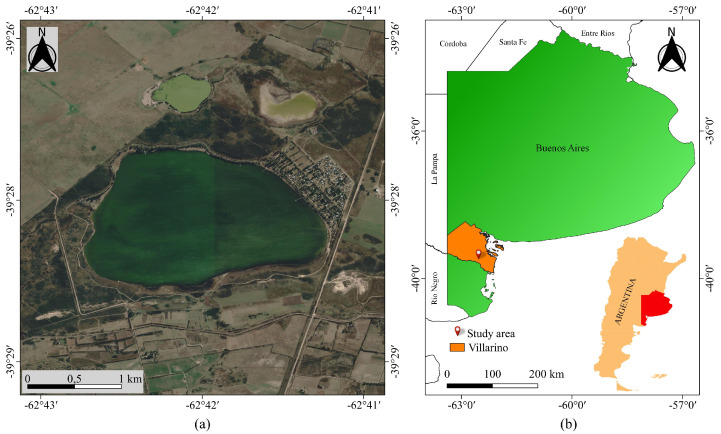
Geographic location of the study area. (**a**) Image of the lake in Google Earth. (**b**) Location of the lake in Buenos Aires Province and in Argentina.

The La Salada lake is located in the South West (SW) of the Buenos Aires province (Argentina) ($39^{{}^{\circ}}28^{\prime }$ S, $62^{{}^{\circ}}42^{\prime }$ O), at about 6 km from the Pedro Luro city (Figure 1). It has an approximate area of 4 km^2^ and a mean depth of 2.5 m. It is a typical endorheic lake where two water channels discharge the excess water from the agriculture usage of the Colorado river basin [18,19]. In the Northeastern portion of the lake, a recreational environment is placed, which is widely forested. This type of small lake is common in the Pampean region, in which sizes and depths of water bodies are dependent on rainfall, presenting periods when they become dry, and other periods with overflow conditions where flooding of the surrounding fields arises. These aspects were extensively confirmed by [20], where it was shown that the topography of the geographic region, the low permeability of the soil, and the shallow character of these lakes cause water to evaporate in periods of drought, reducing its area, and, on the other hand, flooding in periods with more than average rainfall.

### 2.2. Data Acquisition

Airborne RGB imagery from an unmanned aerial vehicle (UAV) and L8 and S2 multi-spectral images were used to carry out this study. Aerial images were acquired within a small time interval with respect to the satellites passing by in a way to avoid temporal variations in the lake that could negatively impact the obtained values. The UAV images were captured on 11 April 2019, at 12 p.m., with optimal atmospheric conditions. For this, a DJI Phantom 3 Standard (DJI, Shenzhen, China) carrying a 12-megapixel camera was used which has a CMOS sensor and a differential GPS sensor. Flight programming and UAV operation were performed using Litchi app installed on a smart device. The images were captured at a height of 70 m for a Ground Sample Distance (GSD) of 2.5 cm, with an overlap of more than 80%. To georeference the photogrammetric model, ten control points were manually selected in the field. The planimetric coordinates were taken using the Real-Time Kinematic (RTK) method in its NTRIP (Networked Transport of RTCM via Internet Protocol) variant. The flight covered only the SW portion of the lake because a safe distance away from the recreational section needed to be respected for legal reasons. With the airborne imagery, a ground truth georeferenced mosaic was created using the Agisoft Metashape Software V1.3.1 (https://www.agisoft.com/, accessed on 11 April 2019). For georeferencing purposes, control points were selected and measured manually. Given that the initial resolution of 2.5 cm/pixel is unnecessary fine for our purposes, and its processing would require large processing times, the mosaic was re-scaled to 15 cm/pixel using the nearest neighbour resampling method.

An L8 georreferenced image of the geographical region was downloaded from the Earth explorer website (https://earthexplorer.usgs.gov/, accessed on 20 March 2023), corresponding to 12 April 2019 (path/row: 226/87) with processing Level-2. Landsat Level-2 products include surface reflectance (Bottom Of Atmosphere—BOA), which measures the fraction of incoming solar radiation that is reflected from the Earth’s surface to the Landsat sensor. Additionally, the Land Surface Reflectance Code (LaSRC) corrects for the temporally, spatially, and spectrally varying scattering and absorbing effects of atmospheric gases, aerosols, and water vapor. This correction is necessary to reliably characterize the Earth’s land surface. A Sentinel-2B satellite image was downloaded from the same website, corresponding to 12 April 2019 (tile number 20HNB) with processing Level-2A. Level-2A products provide scene classification and atmospheric correction, and Bottom Of Atmosphere (BOA) reflectance images derived from the associated Level-1C orthoimage products. In other words, a Level-2A product is an orthoimage with atmospheric correction, resulting in a Surface Reflectance product. The satellite images and the mosaic were georeferenced using the same reference system.

### 2.3. Data Processing

**Figure 2:**
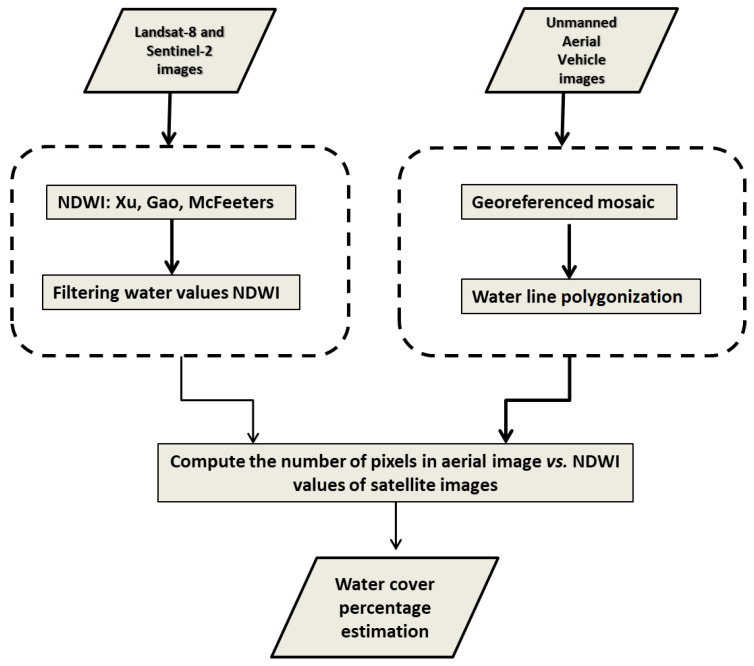
Processing pipeline to estimate water cover percentage in satellite imagery and airborne images.

The processing workflow is composed of three major steps (the complete pipeline is shown in Figure 2).

**Figure 3:**
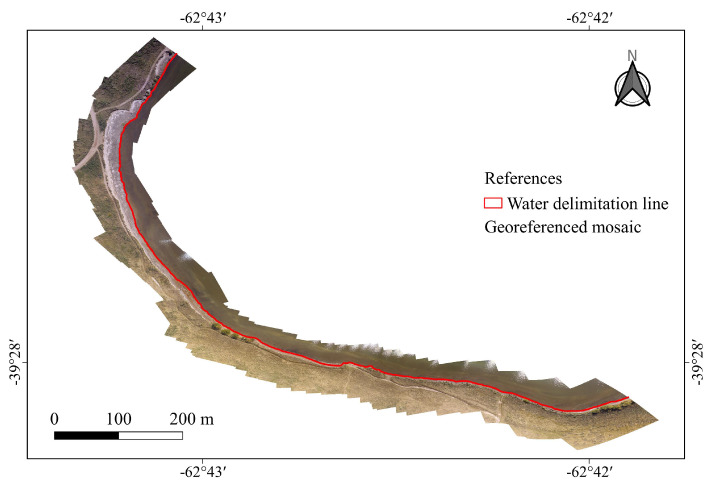
Lake shoreline segmented by expert geographers (in red).

1. The lagoon shoreline is segmented by specialist geographers over the ground truth mosaic (Figure 3). The resulting shapefile is overlapped over the registered Landsat-8 and Sentinel-2 images to establish a set of boundary pixels in each satellite image.
2. Different NDWI models are computed using L8 and S2 images specifically for the mixed pixels, i.e., those arising over the vectorized shoreline using two different criteria (nearest neighbor and linear reconstruction).
3. Actual water cover percentage over the satellite mixed pixels is estimated with the ground truth mosaic, and the correspondence of these percentages with the corresponding NDWI values are represented together.

Three NDWI indices (Xu, Gao, McFeeters) were computed to assess their performance in mixed pixels. McFeeters [21] proposed a NDWI using green and NIR bands given that water generates high reflectance in the green band and low reflectance in the NIR band (Equation (1)). This index can effectively detect and quantify the presence of water in most cases. It has the drawback that it is sensitive to built-up areas and can lead to an overestimation of the size of water bodies.

(1) $$NDWI_{McFeeters}=\frac{\rho_{green}-\rho_{NIR}}{\rho_{green}+\rho_{NIR}}.$$

The method proposed by Xu is similar to that of McFeeters, but uses the SWIR band instead of the NIR band (Equation (2)). This index detects the presence of water features while being insensitive to built-up areas, as well as vegetation and soil-induced noise [22].

(2) $$NDWI_{XU}=\frac{\rho_{Green}-\rho_{SWIR1}}{\rho_{Green}+\rho_{SWIR1}}.$$

**Figure 4:**
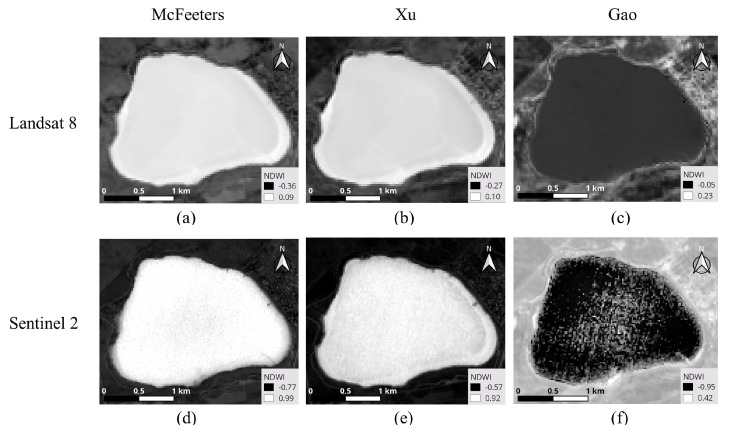
Landsat-8 (upper row) and Sentinel-2 (lower row) NDWI indices: (**a**) L8 McFeeters. (**b**) L8 Xu. (**c**) L8 Gao. (**d**) S2 McFeeters. (**e**) S2 Xu. (**f**) S2 Gao.

Gao proposed another index that is sensitive to changes in the water content of leaves [23]. It is computed using the NIR and SWIR bands (Equation (3)):

(3) $$NDWI_{GAO}=\frac{\rho_{NIR}-\rho_{SWIR1}}{\rho_{NIR}+\rho_{SWIR1}}.$$

where $\rho_{green}$, $\rho_{NIR}$, $\rho_{SWIR1}$ are the reflectance of each bands. These NDWI indices were computed using the raster calculator of QGIS, a free and Open Source Geographic Information System (https://qgis.org/es/site/, accessed on 1 May 2023). The L8 NDWIs were processed using Bands 3 and 6 in Xu, Bands 5 and 6 in Gao and Bands 3 and 5 in the McFeeters index, respectively. In S2 images, Bands 3 and 11 (10 m and 20 m spatial resolution, respectively) were used to compute Xu NDWI; in this case, Band 3 was resampled to have the same spatial resolution as Band 11. For Gao’s NDWI Bands 8A and 11 and for McFeeters’s NDWI Bands 3 and 8 were used (Figure 4).

**Figure 5:**
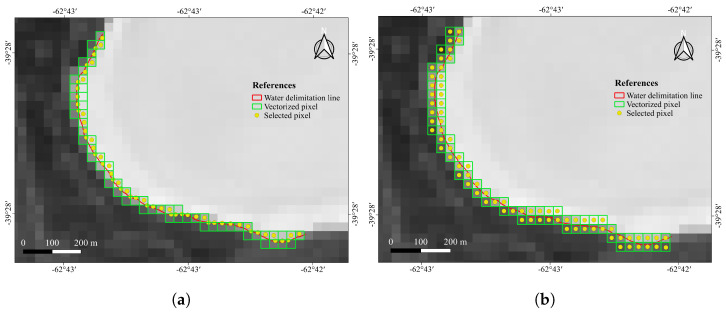
Mixed pixels in L8 Xu index image. (**a**) Zero-order criterion. (**b**) First-order criterion.

**Figure 6:**
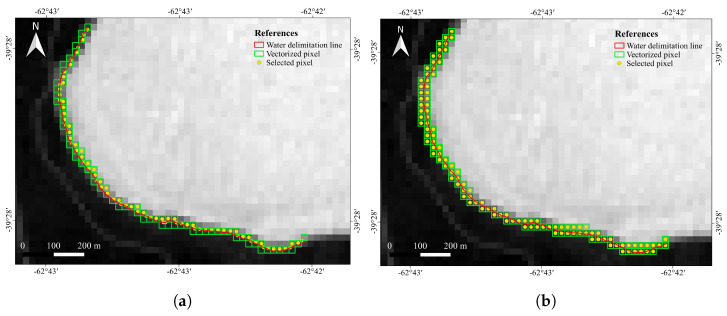
Mixed pixels in the S2 Xu index image. (**a**) Zero-order criterion. (**b**) First-order criterion.

The actual shoreline (mixed) pixels in the satellite images were determined according two different criteria. The first criterion is the modeling of the pixel acquisition as a zero-order process, and therefore the pixel with its center closest to the segmented shoreline is considered to be the raster representation of the shoreline in the satellite image. This criterion is coincident with the so-called nearest neighbour in image processing. The second criterion is the assumption of a first-order image generation process during satellite acquisition. According to this criterion, the actual vectorized shoreline passes between two pixel centers, and therefore these two pixels are, in fact, mixed pixels to some extent (unless the very rare case in which the vectorized shoreline passes exactly over the very center of one pixel). Figure 5 and Figure 6 show these two criteria applied, respectively, over the Landsat and Sentinel images.

**Figure 7:**
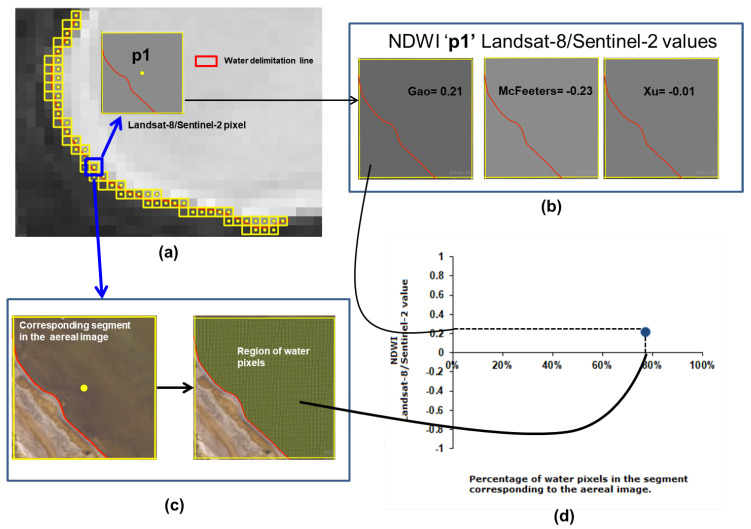
Overview of the method. (**a**) For each pixel in the contour (highlighted in blue), the NDWI is computed with the selected method. (**b**) The footprint of the pixel is put into correspondence with the aerial image and the percentage of water pixels therein is computed. (**c**) These two values (NDWI and water pixel percentage) determine the coordinates of the given pixel. (**d**) Scatterplot of NDWI value and water percetage in each pixel of the aerial image.

Each pixel footprint was aligned with the ground truth mosaic (where they occupy hundreds or thousands of pixels, according to their respective resolutions), and, subsequently, the actual percentage of water pixels in the mosaic was computed. With these two values for each satellite shoreline pixel corresponding, a scatterplot was built to test the actual correlation between the NDWI and the actual percentage of water according to the ground truth; then, the actual correspondence between NDWI and actual water coverage could be assessed (Figure 7). This gave rise to four different scatterplots for each of the NDWI indices corresponding to each of the satellite missions and shoreline determination criteria.

## 3. Results

### 3.1. Landsat-8 and Sentinel-2 Zero-Order Criterion

**Figure 8:**
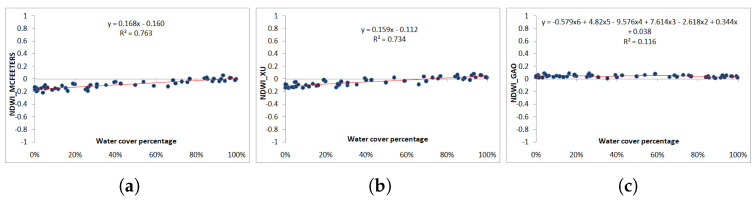
Water cover percentage and NDWI values obtained from the vectorized pixels according to zero-order criterion using Landsat-8 image. (**a**) McFeeters NDWI. (**b**) Xu NDWI. (**c**) Gao NDWI.

Figure 8a shows the NDWI values according to the McFeeters index. Values are mostly negative (in the range of [−0.21, 0.05]) and the regression coefficient is the highest ($R^{2}$ = 0.763). Only a few positive NDWI pixels are actually covered by a higher water percentage as per the ground truth. Xu’s proposal generates similar NDWI values (in the range of [−0.14, 0.08]) and the regression coefficient is also similar to that of McFeeters ($R^{2}$ = 0.734) (Figure 8b). In both plots, the regression line has a low positive slope, showing a slight positive correlation between the computed NDWI value and the percentage of water in the analyzed pixels. The NDWI values obtained from Gao’s equation are all positive [0, 0.09], regardless of pixel water coverage (Figure 8c). The regression line is almost horizontal, with a lower regression coefficient ($R^{2}$ = 0.116), and NDWI values are similar for pixels with either low or high water coverage.

**Figure 9:**
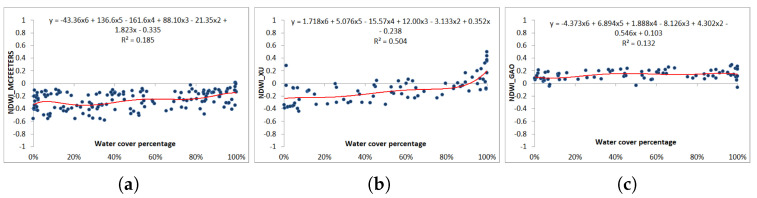
Water cover percentage and NDWI values obtained from the vectorized pixels according to zero-order criterion using Sentinel 2 image. (**a**) McFeeters NDWI. (**b**) Xu NDWI. (**c**) Gao NDWI.

For Sentinel-2 images, the Xu NDWI values are mostly negative (72%). Few pixels with positive NDVI values are covered by a significant proportion of water, between 40% and 100% (Figure 9b). The regression coefficient is the highest ($R^{2}$ = 0.504). McFeeters NDWI values are all negative (98%), and few pixels with positive values are covered by a large percentage of water (higher than 90%) (Figure 9a). Gao’s NDWI values are mostly positive (95%), regardless of pixel water coverage (Figure 9c). The correlation function in the three plots are fitted to polynomial functions of the order of six, since these were the functions which achieved the best regression coefficient. In these two latter models, the regression coefficient is rather low ($R^{2}$ = 0.185 and 0.132, respectively).

### 3.2. Landsat-8 and Sentinel-2 First-Order Criterion

**Figure 10:**
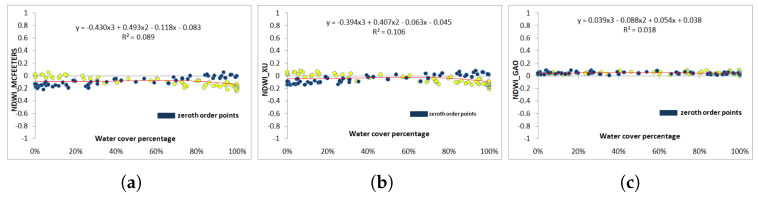
Water cover percentage and NDWI values obtained from the vectorized pixels according to the first order using Landsat-8 image (in yellow). (**a**) McFeeters NDWI. (**b**) Xu NDWI. (**c**) Gao NDWI. The zero-order values are represented in blue.

**Figure 11:**
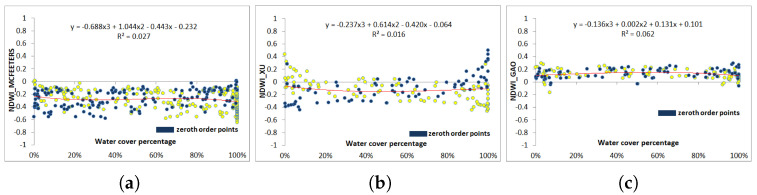
Water cover percentage and NDWI values obtained from the vectorized pixels according to the first order using Sentinel 2 image (in yellow). (**a**) McFeeters NDWI. (**b**) Xu NDWI. (**c**) Gao NDWI. The zero-order values are represented in blue.

Figure 10 and Figure 11 show the scatterplots computed using the first-order criterion (in yellow), where the zero-order values are represented in blue for comparison reasons. In the six cases (L8 and S2 for the three NDWI models), the regression coefficients are below 0.1, meaning that the actual influence of the water coverage in the mixed pixels has almost no influence in the computed NDWI. With L8 images, Figure 10b shows that Xu’s values are mostly negative (70%), and the few positive NDVI pixels have indistinctly low or high water coverage. McFeeters’s NDWI values are mostly negative (87%) (Figure 10a). Gao’s NDWI values are all positive, between 0.01 and 0.09 (Figure 10c). The fitting correlation function in the three cases is a third-order polynomial function, which, again, yields the highest regression coefficient.

For S2 images, Figure 11b shows that Xu’s NDWI values are mostly negative (77%), whereas a few pixels with negative values are those with the greatest water coverage. On the contrary, McFeeters values are all negative, between −0.64 and −0.01 (Figure 11a). As with the zero-order criterion (Figure 11c), Gao NDWI values are mainly positive with values between −0.17 and 0.29, regardless of pixel water coverage. The regression function in the three plots is fitted to a polynomial function of the order of three. It can also be noticed that S2 values are much more dispersed with respect to the fitting function compared with L8 values.

## 4. Discussion and Conclusions

This research aimed to assess the quality of NDWI values and their correlation with pixel water coverage in a small water body that is representative of similar lakes and lagoons that are common in the SW of the Buenos Aires province (Argentina). Twelve different evaluations were performed (three indices, two satellite constellations, and two criteria for shoreline pixel selection). The results show that these values in the two most widespread satellite constellations and using three different NDWI formulations do not have the expected accuracy and consistency. In general, the results show a bias towards negative NDWI values, which is not a problem by itself, given that the indices require an interpretation in order to be applied. Also, the fitting models (computed NDWI vs. actual water coverage) were much more dispersed when using S2 images compared with the cases when L8 was used. This result implies that for this specific land cover determination problem (shallow inland waters, small water bodies, shoreline determination, etc.), L8 provides more robust and consistent determinations. Therefore, a trade-off exists between this consistency and the coarser spatial resolution when compared with S2 images. Regarding the shoreline reconstruction criteria, in all cases, the zero order exhibited a stronger correlation. This is counter-intuitive since the actual imaging process is indeed a first-order reconstruction of the actual Earth backscatter. This criterion selects as mixed pixels several pixels with a very high water coverage in the ground truth, but, as shown in Figure 10 and Figure 11, the associated NDWI values are not high in these pixels (and are significantly below the mean in S2 images). This can be attributed to differences in the actual spectral bands of the sensors, and that the spectral bands used by Sentinel sensor are more affected by the water turbidity than with the Landsat sensor.

Xu’s NDWI values were consistently more correlated with the actual water coverage of the image pixels in all evaluations. This index presented a moderate positive correlation to the actual water coverage of the pixels in the zero-order shoreline reconstruction method, and a negative correlation in the second criterion. The values themselves were mostly negative for the Landsat-8 image. This may be attributed to the fact that Xu’s equation has been shown to be effective on a wide range of sensors, and different studies show that this index is accurate in water bodies with a shallow depth, in areas with high turbidity, or where shallow waters are vegetated [24]. The NDWI index proposed by Gao shows a tendency to overestimate the water covering percentage, detecting the presence of water in bare ground and soil with vegetation. Compared to the other indices, it shows the less accurate results, both for L8 and S2 images, and in both criteria. It is likely that the spectral bands in the index computation are not sensitive to the variation of the reflectance of the water in relation to the reflectance of the surrounding soil in this particular geographic context. Finally, NDWI values obtained by the McFeeters index are almost always negative, for both images and criteria. In the case of L8 images, this underestimation of the water coverage is weaker, and for S2 images, the underestimation is stronger.

Since flood maps computed from satellite images almost in real time are becoming a vital tool for decision-making in contingency management and disaster monitoring and evaluation, the choice of imagery and index (and also the interpretation of the indices themselves) should be taken with special care. Flooded areas contain mixtures of water and land, vegetation, or even urban areas. Therefore, only a fraction of the pixel is free water, which in turn may be turbid or carry a large amount of material in suspension. This contribution shows that the choice of one index over another depends on the specific characteristics of the satellite image and the water body being studied.

## Data Availability

Not applicable.
